# Assessing Training Practices and Gaps for Staff Involved in the Delivery of Oncology Financial Navigation: A Qualitative Study

**DOI:** 10.3390/curroncol33020130

**Published:** 2026-02-23

**Authors:** Gaby Cordero, Maria Pisu, Shu-Fan Chen, Elizabeth Ward, Margaret I. Liang

**Affiliations:** 1 Jacobs School of Medicine and Biomedical Sciences, University at Buffalo, Buffalo, NY 14203, USA; 2 Division of General Internal Medicine and Population Science, and O’Neal Comprehensive Cancer Center, University of Alabama at Birmingham, Birmingham, AL 35233, USA; 3 Department of Obstetrics & Gynecology, Cedars-Sinai Medical Center, Los Angeles, CA 90048, USA; 4 Alabama College of Osteopathic Medicine, Dothan, AL 36303, USA; 5 Division of Gynecologic Oncology, Department of Obstetrics & Gynecology, and O’Neal Comprehensive Cancer Center, University of Alabama at Birmingham, Birmingham, AL 35233, USA

**Keywords:** financial navigation, financial hardship, financial toxicity, costs of cancer care, cancer supportive services

## Abstract

Financial hardship affects 30–70% of cancer patients and is associated with worse quality-of-life outcomes and higher mortality. In response, many health systems have implemented financial navigation teams to mitigate financial hardship and provide financial guidance to cancer patients. Currently, there is a lack of standardization in financial navigation training. Our primary objective was to assess current training practices and gaps that may exist in critical information and tools for day-to-day operations for individuals providing financial navigation services. Our secondary objective was to supplement findings from the interviews with a web-based search for training resources that would be helpful in these roles. Semi-structured qualitative interviews were conducted over a video-based conferencing platform in the United States of America with nine individuals in varying roles related to financial navigation. Thematic analysis was conducted by investigators to identify common themes using a constant comparative method. Current financial navigation training practices were found to be less structured and comprehensive than desired, largely relying on experiential “on the job” learning. Participants expressed the need for more multi-dimensional training that covers insurance, cancer treatment and associated costs, financial resources, and an emphasis on developing soft skills to navigate the sensitive topics of cancer and cancer costs. The findings contribute to the development of more standardized trainings that incorporate dissemination of crucial financial information in a compassionate manner. A web-based search was also performed to create a compilation of available financial navigation training resources.

## Simple Summary

Financial hardship is extremely common in cancer patients and can cause delayed treatment and worse health outcomes. Although some health systems employ financial navigation teams to help patients with financial hardship, there is currently no clear standard for training these teams. In this study, we conducted interviews with individuals in financial navigator roles to better understand current training practices and performed a web search to assess current training resources. Participants expressed that most jobs relied on “hands-on” training and that there was a need for more structured and comprehensive training that covers both relevant financial topics and soft skills to communicate with patients on this sensitive topic.

## 1. Introduction

A cancer diagnosis can place patients at risk for financial hardship due to accumulation of out-of-pocket costs and decreased ability to continue working for income while undergoing treatment [1,2,3,4]. Studies show that financial burden affects 30–70% of cancer patients and can lead to psychological distress, negatively impact patients’ material conditions, and result in health-related coping behaviors like delaying care or not taking medications as instructed due to costs [1,5]. To address this problem, health systems have implemented financial navigation teams, which are defined by the National Cancer Institute (NCI) as teams that work “with patients and their families to help them reduce stress or hardship related to the cost of treatment for a medical condition, such as cancer” [6].

Financial navigation comprises three core functions, which include (1) preparing patients for out-of-pocket medical costs, (2) optimizing health insurance, and (3) screening patients for financial concerns and linking them to available financial assistance programs [6,7,8]. Financial navigation teams are multidisciplinary and can involve many different roles. Depending on the setting and available personnel, these team members can include social workers, financial counselors, patient navigators, billing staff, pharmacy staff, and oncology providers [9]. As these roles are increasingly needed across cancer centers, it is important to understand what training is needed for these care team members to best address patients’ financial needs [10]. To address this knowledge gap, we conducted qualitative interviews with nine individuals at seven institutions involved in financial navigation. Our primary objective was to assess current training practices and gaps that may exist in critical information and tools for day-to-day operations for individuals providing financial navigation services. Our secondary objective was to supplement findings from the interviews with a web-based search for training resources that would be helpful in these roles.

## 2. Materials and Methods

The current study was approved by the University of Alabama at Birmingham Institutional Review Board (IRB #300006699). We conducted qualitative interviews with participants involved in financial navigation services from diverse cancer centers across the country and performed a web-based search of available training resources.

### 2.1. Participants and Recruitment

We recruited a convenient sample of participants from a purposeful selection of cancer centers which were diverse in terms of hospital size (based on number of hospital beds), geographic location (West, Midwest, Northeast, Southwest, Southeast), and practice type (county safety net hospital, NCI-designated Comprehensive Cancer Center, NCI-designated Cancer Center, community not-for-profit hospital, community for profit hospital). Potential participants were eligible if their role included helping patients address financial needs related to their cancer care. They were contacted via electronic mail and were provided with an IRB-approved information sheet that explained the purpose, procedures, risks, and benefits of their voluntary participation in this study. If potential participants felt there was a more appropriate or available interviewee, they would refer that individual to the study team. The information sheet was reviewed before the interview and verbal consent was obtained. There was no incentive provided for participating in the study.

### 2.2. Conduct of Interviews

**Figure 1:**
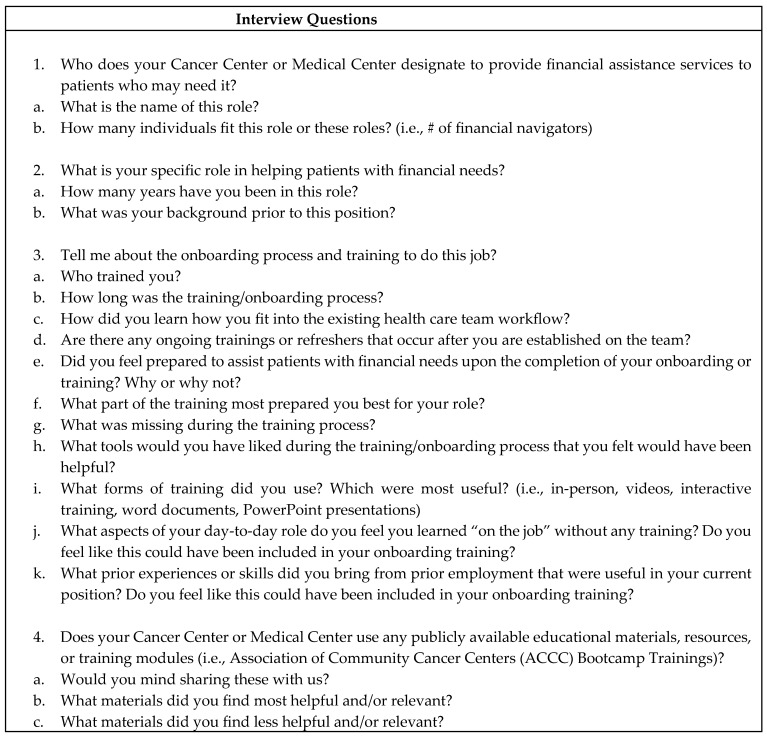

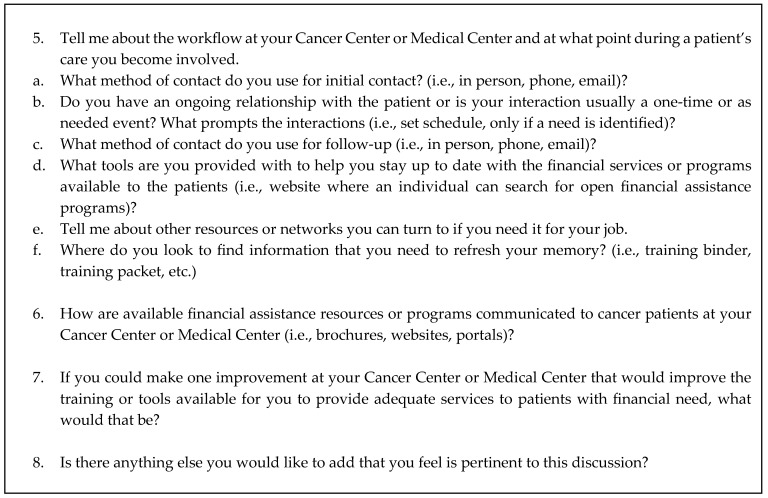
Semi-structured interview guide for individuals providing financial navigation services to oncology patients.

We conducted 30 minute semi-structured interviews using an interview guide (Figure 1) in Summer 2021. Our two main constructs were (1) current onboarding and training and (2) critical knowledge base needed and tools available for day-to-day operations. Question probes asked about financial assistance services, the interviewee’s specific role and experience, onboarding and training processes, publicly available training resources, day-to-day workflow including knowledge and tools used, communication with patients, and desired improvements. The interview guide was created by three investigators (G.C., M.P., M.I.L.) and training in qualitative interview techniques was provided by two investigators (M.P., M.I.L.). One investigator (G.C.) conducted all the interviews on a secure video-based conferencing platform.

### 2.3. Analysis

The video-based interviews were recorded, and the audio was transcribed verbatim by an independent commercial transcription company. Thematic analysis was conducted by the investigators (G.C., M.P., M.I.L.) to identify common themes using a constant comparative method. Each participant’s responses were evaluated at an individual level and weighted equally rather than taking the size of the health system they worked at into account.

### 2.4. Web-Based Search of Existing Training Resources

Three investigators (G.C., E.W., S.C.) searched the web using the following terms: “financial counseling”, “financial navigation training”, “health insurance training”, and “training cancer center” to find existing financial counselor training resources. When not promptly available, we contacted companies offering training resources to assess potential costs of implementing their advertised training and resource products (i.e., Navectis). In addition, the investigators explored online forums (i.e., ACCCeXchange, a members-only virtual discussion forum for individuals involved in financial navigation) that interviewees shared were beneficial in their training processes.

## 3. Results

### 3.1. Participant Characteristics

At least one individual from all seven sites that were contacted agreed to participate. In some cases, an individual referred the investigators to a more appropriate or available interviewee. Study participants included nine interviewees from a total of seven cancer centers. Characteristics of participants and their institutions are summarized in Table 1. Participant roles at their institution included care navigators, social workers, financial counselors, financial analysts, and patient navigators. The average time in their current financial navigation role was three years (range 1–23). The seven cancer centers varied in size from 300 to 1200 hospital beds. There were several institution types represented, including three NCI-designated Comprehensive Cancer Centers and two community not-for-profit hospitals.

### 3.2. Construct 1. Onboarding and Training

#### 3.2.1. Theme 1: Learning on the Job

We found that a large portion of the responsibilities of the role and how to fulfill them was learned on the job. Participants felt their job responsibilities were not always clearly delineated upon starting their position. One financial counselor shared, “It was a learn as you go situation…one day at a time…going through the information, and what was needed for the position” [3 years in role]. Many found that shadowing a previous employee in a similar role was the most helpful, particularly to learn institutional and state-specific processes. A social worker commented, “I got to go in and actually watch another social worker interact with these patients…I think that’s the most beneficial thing” [3 years in role].

#### 3.2.2. Theme 2: Drawing on Previous Work Experience

Many of the participants were being hired into a position that was recently or newly created and found it was important to use knowledge from past employment. For example, a patient care management educator shared it was helpful “having an oncology background, but I also worked in public health in rural communities… understanding the needs at a community level and working with the resources directly at that level was definitely helpful coming in” [4 years in role]. Participants came from former positions that included accounting and public health or from settings such as the hospital emergency room and outpatient services. One lead oncology financial analyst commented, “My background as a certified public accountant and I was a bank manager… I know how to deal with these reports and how to develop the work” [1 year in role].

#### 3.2.3. Theme 3: Creating Self-Taught Training Guides

There was typically no formal training guide and individuals in these roles would search for guidance on the internet when trying to learn specific aspects of their job. Generally, participants described their experience with onboarding as a self-taught experience. For example, one financial counselor described their first few months in the role, “We had to basically create everything for the role,” [3 years in role] and a care navigator stated, “I would have calls [with other cancer center sites] and ask them a lot of questions. And I created that as my own training” [2 years in role]. Many participants described creating their own learning guides through their accumulated experiences and prior jobs to guide their current positions.

#### 3.2.4. Theme 4: Using Available Online Training Resources

The most used resource for onboarding and training came from the Association of Cancer Care Centers (ACCC) financial advocacy bootcamp training. The program provides training on fundamentals of financial advocacy, enhancing communication with patients, background on oncology, and tools to screen patients for financial distress. One financial counseling manager shared, “ACCC is probably the best one out there… we also use Triage Cancer” [2.5 years in role].

### 3.3. Construct 2: Critical Knowledge Base and Tools for Day-to-Day Operations

#### 3.3.1. Theme 1: Fundamental Knowledge of Insurance, Drugs, and Oncology Needed

Knowledge of insurance, prescribed drug costs, oncology terminology, and common treatment plans is crucial to include in the onboarding process and day-to-day functions of individuals providing financial navigation. This knowledge provides a foundation for assistance to be tailored to each patient’s specific cancer treatment and insurance situation. One manager of financial counseling shared, “Benefit verification is very important when it comes to helping patients understand their out-of-pocket costs” [2.5 years in role]. Many of the participants benefited from their prior knowledge of insurance, such as Medicaid, to successfully do their job, but noted that official training would have been helpful. Given the complexity of cancer care, a lead oncology financial analyst stated, “I learned about chemotherapy [on the job] a lot… about the diagnosis and costs, and I didn’t know about these codes, [or] how to deal with the insurances,” [1 year in role] and a financial counselor commented, “There are so many drugs. I don’t understand a lot of it. I just know I have to look it up” [3 years in role].

#### 3.3.2. Theme 2: Soft Skills Training Desired

Formal training on soft skills (e.g., communication and interpersonal skills) to navigate difficult patient conversations was deemed helpful and desired. Participants acknowledged that patient conversations in oncology can present unique challenges for the team providing care. One social worker described a challenging interaction that they did not feel adequately trained to navigate, “I’ve had patients call me and ask me for 20 bucks. I mean, that’s one of the things that’s included in boundaries. What do you do when a patient calls you and asks you for 20 bucks?” [3 years in role]. Another social worker described the usefulness of soft skills in interacting with patients, “It’s people skills, it’s learning how to ask those difficult questions. It’s learning how to build rapport; it’s learning how to really get in and identify some of those needs. And, those are really softer skills” [11 years in role].

#### 3.3.3. Theme 3: Organizational Tool Often Manually Created

Similarly to Theme 3 for Construct 1, a comprehensive set of tools available and ready to use are needed but often must be created manually. Examples shared by a financial counselor included, “I had a binder that I kept all my information in and…I could flip back through to help with certain information [or find] forms” [3 years in role] and by a lead oncology financial analyst included, “I built my own spreadsheets…with formulas to keep track of billing and claim checks received by different foundations” [1 year in role]. Another participant described having to reach out to individual organizations to find specific support programs for their patients. A care navigator reported, “I’m printing out the specific organizations, their information, so that [when] I meet with the patient, we can then call these organizations, or we can work together to get these services” [2 years in role].

The constructs and themes above are summarized in Table 2.

### 3.4. Web Search for Financial Navigation Resources

Through our qualitative interviews and supplemental web search, we identified a sample of resources that can be used to train or support staff in financial navigation roles. The search was last updated in July 2025. Resources are summarized in Table A1 and include both free and paid services ranging from online training modules, search tools for financial assistance programs, insurance resources, and programs that integrate into the electronic health record to estimate patient out-of-pocket costs. We found six financial navigation training programs, with four available for free and two requiring payment to access training materials. Some trainings were more involved, requiring in-person participation, while others consisted of self-paced trainings and self-evaluations. Common training topics included understanding insurance, finding external assistance, patient advocacy, and empathetic communication. We outline eight helpful and common tools for financial navigation, all of which were free to use. Many of these were also mentioned by the interviewee participants. The tools were largely either resource directories, direct financial assistance, or education for cancer patients.

## 4. Discussion

Given the rising incidence of early-onset cancer in people under age 50 and improving 5-year survival rates reaching 70% for all cancers combined, more patients are living longer with cancer [11,12]. In addition, trends such as overall rising costs of cancer care, increased patient cost-sharing, and underinsurance are contributing to the financial burden that cancer patients and their caregivers face [13,14]. Thus, proactively assessing financial navigation capacity for cancer patients is more crucial than ever. Our study contributes new information on the current landscape of onboarding and training and the necessary knowledge and tools from the perspective of individuals in financial navigation roles at diverse cancer centers across the country. Most participants expressed the need for more structured training for individuals in these roles. Experiential on-the-job training was the most common reported training method, and participants frequently reported using financial knowledge or skills from prior positions to navigate their current responsibilities. Participants also created their own organizational tools to manage workflows and to identify and organize available financial assistance programs. A leading resource used in training and supporting financial navigation team members was through the ACCC Financial Advocacy Network.

Our findings are consistent with reports that demonstrate a need for more structured onboarding and training for financial navigators. A 2021 NCI survey distributed to 63 cancer centers reported that about 40% of respondents agreed that staff are not equipped to discuss financial issues with cancer patients and agreed that staff lack awareness about available financial navigation services for these patients [9]. A recent study of over 500 participants (51% oncology financial advocates and 42% mix of patient navigators, nurse navigators, social workers, pharmacists, advanced practice providers, and nurses) analyzed the effectiveness of the ACCC Financial Advocacy Bootcamp self-guided training program for oncology financial advocates by comparing pre- and post-training knowledge and skills [15]. Their findings demonstrated significant self-reported improvement in participants’ ability to incorporate effective screening methods to identify patients at risk of financial toxicity, review public and private health insurance, and find patient resource and assistance programs. Training resources like those provided by the ACCC serve to equip financial personnel with general strategies and resources to help alleviate patient financial hardship. Outlining prior work experiences that have contributed to success in financial navigation roles and utilizing structured training can provide a framework as cancer programs develop the infrastructure for financial navigation. Financial navigation overlaps with but is distinct from oncology patient navigation; however, existing job descriptions for patient navigators can be useful to adapt [16].

While resources exist to help create and support financial navigation programs, the act of finding relevant resources is extraordinarily complex. As noted by our interviewees, the specific cancer diagnosis (e.g., lymphoma vs. breast cancer), patient circumstance (e.g., specific financial need or patient household income level), and setting (e.g., institution or geographic region) may have specific financial assistance programs available, which often requires manual searching and organization of available resources. Further work should be conducted to develop a toolkit that can be customized to each setting; for instance, a method to organize assistance programs based on common patient eligibility criteria. Some interviewees reported using online search engines like Google to look up resources related to care when they did not know where to turn. Furthermore, insurance concerns are a common barrier for patients who are newly diagnosed with cancer and trying to navigate their coverage [17]. Our interviews found that it is essential for financial navigators to be well versed in the fundamentals of insurance, prescribed drug costs, and oncology terminology. Some interventions to provide this information are being developed and tested: for example, the HIAYA CHAT (Huntsman Intermountain Adolescent and Young Adult Cancer Care Program ‘Let’s chat about health insurance’) intervention is aimed at increasing health insurance literacy and decreasing financial distress among adolescent and young adult cancer patients. The training for the patient navigator delivering the HIAYA CHAT intervention incorporates training from George Washington Cancer Center, Triage Cancer, and ACCC, which are also included in the resources we describe in Table A1 [18,19]. Whether these resources are sufficient for navigators to be comfortable addressing insurance complexities and talking about costs of care remains to be established. The Lessening the Impact of Financial Toxicity (LIFT) intervention is a financial navigation intervention designed to reduce financial toxicity [20]. Training of financial navigators delivering the LIFT intervention also involved the ACCC Financial Advocacy Bootcamp as well as other topics mentioned by our interviewees, including information on cancer care trajectories and review of financial resources. Additional training further emphasized in the training of LIFT financial navigators included case management skills (e.g., building and using case tracking tools) and having access to more experienced individuals who could help train and mentor individuals newer to the role [20].

The most desired skills training shared by interviewees was learning how to conduct sensitive conversations about financial topics sympathetically and privately with cancer patients. One qualitative study of real clinical encounters identified key aspects of communication to support patient financial capacity such as asking questions, being kind and acknowledging emotions, noting indirect signals from patients, and making cost conversations a standard part of clinical care [21]. In another study, breast cancer survivors and care team members highlighted the sensitive nature of cost of care conversations, with essential elements including reassurance that costs will not prohibit the receipt of recommended care and a discussion of an action plan for patients to be able to afford their treatment [22]. In addition, extrapolating communication training from other disciplines can be useful. Institutions have successfully trained lay navigators to assess patient distress appropriately and help them find solutions to challenges like transportation [23], a process that aligns with the goals of financial navigation.

A strength of this study is the variety of perspectives of our interviewees who worked at cancer centers differing in institution type, size, and geographic location. Additionally, our interviewees had different titles and roles which further represents the diversity of practice contexts and provided us a broad perspective on financial navigation services. The primary limitation of the current study is the small sample size and convenience sampling, which may not make our findings generalizable to all oncology practices.

Continued development and information sharing of standardized financial navigation training will improve the availability and effectiveness of financial navigation services and help to ensure all cancer patients receive an equal opportunity to have their financial needs identified and met. There are existing resources for cancer programs and practices to continue to build and expand financial navigation capacity. Core functions of financial navigation have been described, including systematic screening and tracking, identifying then responding to patient-specific needs, developing strong relationships, and actively removing common barriers [20]. In addition, the ACCC Financial Advocacy Network has developed a Financial Advocacy Services Assessment tool for practices to assess, monitor, and improve the delivery of their financial advocacy services [24]. Effective onboarding trainings must include multidimensional components that prepare these team members for discussions on insurance, treatment costs, accessing financial assistance resources, and the soft skills needed to effectively communicate with patients on the sensitive topic of the costs of cancer care while balancing compassionate, yet informative care.

## Figures and Tables

**Table 1:** Characteristics of participants and their health care institutions.

**Health System Characteristics (*n* = 7)**	
Size of hospital (median, range)	500 (300–1200)
Type (*n*, %) NCI- Designated CCC Community, not-for-profit hospital County safety net hospital	4, 57% 2, 29% 1, 14%
Geographic regions Northeast Southeast Southwest Midwest West	2, 29% 2, 29% 1, 14% 1, 14% 1, 14%
**Participant Characteristics (*n* = 9)**	
Positions represented	Educator for Patient Care Management (*n* = 2) Patient Navigator (*n* = 2) Lead Oncology Financial Analyst (*n* = 1) Director of Social Work (*n* = 1) Social Worker (*n* = 1) Manager of Financial Counseling (*n* = 1) Financial Counselor (*n* = 1)
Years in current position (median, range)	3 (1–11)
Length of time at current institution in years (median, range)	10 (1–23)

NCI = National Cancer Institute; CCC = Comprehensive Cancer Center.

**Table 2:** Themes related to (1) onboarding and training and (2) knowledge and tools for individuals in financial navigation roles.

**Construct 1: Onboarding and Training**
**Theme 1: Learning on the job.**
*It was a learn as you go situation…one day at a time… going through the information, and what was needed for the position.* [financial counselor, 3 years in role]
*I got to go in and actually watch another social worker interact with these patients…* *I think that’s the most beneficial thing.* [social worker, 3 years in role]
**Theme 2: Drawing on previous work experience.**
*Having an oncology background, but I also worked in public health in rural communities… understanding the needs at a community level and working with the resources directly at that level was definitely helpful coming in. [educator of patient care management, 4 years in role]*
*My background as a certified public accountant and I was a bank manager… I know how to deal with these reports and how to develop the work.* [lead oncology financial analyst, 1 year in role]
**Theme 3: Creating self-taught training guides.**
*We had to basically create everything for the role.* [financial counselor, 3 years in role]
*I would have calls [with other cancer center sites] and ask them a lot of questions. And I created that as my own training. [care navigator, 2 years in role]*
**Theme 4: Using available online training resources.**
*ACCC is probably the best one out there, but we also use Triage Cancer.* [manager of financial counseling, 2.5 years in role]
**Construct 2. Critical knowledge base and tools for day-to-day operations**
**Theme 1: Fundamental knowledge of insurance, drugs, and oncology needed.**
*Benefit verification is very important when it comes to helping patients understand their out-of-pocket costs.* [manager of financial counseling, 2.5 years in role]
*I learned about chemotherapy [on the job] a lot… about the diagnosis and costs, and I didn’t know about these codes, [or] how to deal with the insurances. [lead oncology financial analyst, 1 year in role]*
**Theme 2: Soft skills training desired.**
*I’ve had patients call me and ask me for 20 bucks. I mean, that’s one of the things that’s included in boundaries. What do you do when a patient calls you and asks you for 20 bucks?* [social worker, 3 years in role]
*It’s people skills, it’s learning how to ask those difficult questions. It’s learning how to build rapport; it’s learning how to really get in and identify some of those needs. And, those are really softer skills.* [social worker, 11 years in role]
**Theme 3: Organizational tool often manually created.**
*I had a binder that I kept all my information in and…I could flip back through to help with certain information [or find] forms.* [financial counselor, 3 years in role]
*I built my own spreadsheets… with formulas to keep track of billing and claim checks received by different foundations.* [lead oncology financial analyst, 1 year in role]
*I’m printing out the specific organizations, their information, so that [when] I meet with the patient, we can then call these organizations, or we can work together to get these services.* [care navigator, 2 years in role]

## Data Availability

The original contributions presented in this study are included in the article. Further inquiries can be directed to the corresponding author.

## Abbreviations

The following abbreviations are used in this manuscript:

IRB	Institutional Review Board
ACCC	Association of Community Cancer Centers
NCI	National Cancer Institute
CC	Cancer Center
CCC	Comprehensive Cancer Center
LIFT	Lessening the Impact of Financial Toxicity

## Appendix A

### Appendix A.1

**Table A1:** Sample training resources and tools for cancer center staff involved in financial navigation roles cited by interview participants or identified by research literature and web search (last updated July 2025).

Name	Description	Topics	Cost
**Training Resources for Role**			
American Cancer Society (ACS): Leadership in Oncology Navigation (LION)	Self-paced, online standardized training and credentialing program that helps navigators deliver professional patient navigation to individuals, caregivers, and families experiencing cancer.	Ten modules: Navigation Basics Cancer Basics Person-centered Assessments Health System Services Access and Coordination Community-based Services Access and Coordination Community Assessment Patient and Family Communication Promoting Patient Self-Advocacy Facilitation of Person-centered Support Professionalism and Ethical Conduct	$495 for 90 days of program access and participation
Association of Community Cancer Centers (ACCC): Financial Advocacy Bootcamp	Self-paced, online training designed to help financial navigators address financial toxicity in cancer care.	1. Financial Advocacy Bootcamp Level I Financial Advocacy Fundamentals Enhancing Communication Improving Insurance Coverage Maximizing External Assistance Developing and Improving Financial Advocacy Programs and Services 2. Financial Advocacy Bootcamp Level II Oncology 101 Financial Distress Screening Cost-Related Health Literacy Measuring and Reporting Outcomes	Free to members of ACCC Membership Options: Cancer Program Membership (yearly) ACCC Individual Memberships: cancer center employees, consulting company employees, pharmaceutical employees, etc.
George Washington Cancer Center—Financial Navigation Lesson for Oncology Patient Navigators	This training describes current evidence on drivers of financial toxicity, shares mitigation strategies and resources that prepare and equip patient navigators to effectively address financial toxicity.	Lessons Financial navigation lesson for oncology patient navigators Financial navigation lesson for social workers Oncology, patient navigator training: the fundamentals Together, equitable, accessible, meaningful team training Advance, equitable, accessible, and patient centered cancer care through improved patient-provider, communication, cultural sensitivity, shared decision-making, and attention to health literacy	Free
NaVectis Group	Educate and train healthcare providers on how to improve the process of patient financial navigation services Training: 1st part: 2 day—16 h of classroom style training on site (12 h theory, 4 h solving case scenarios) 2nd part: 12 month remote mentorship Remote: email, phone, webex call Can discuss specific patient cases to determine options to decrease financial burden/toxicity by e-mail, phone, Webex call	Training topics: Utilizing Pharmaceutical Free Drug programs (learning when and when not to use these programs) Maximizing Co-Pay assistance programs Utilizing Premium Assistance Programs (internal and external resources) Review of Medicaid options Maximizing the Affordable Care Act Maximizing Medicare	Paid resource for health systems: base Charge—to train 1 financial navigator and provide 12 months mentorship
Patient Advocate Foundation (PAF)—Website Training	Resources for patient and patient counselors related to co-pay relief, financial aids, scholarships. Education Resource Library includes educational videos on choosing a healthcare plan, including education of Medicaid. National Financial Resource Directory Tour helps website users understand purpose and intent of the National Financial Resource Directory financial navigation tool	Training Topics: An Overview of the U.S. Healthcare System Individual and Employer-Sponsored Health Insurance Government Sponsored Health Insurance Tips on Using Health Insurance and Appeals Navigating Disability Insurance and Appeals Managing Finances and Getting Financial Help	Free with e-mail sign up Free training webcast for patients, caregivers, advocates, and providers. Registration required.
Triage Cancer/Triage Health Finance Intensive Training Program	Training program designed specifically for oncology health care professionals and advocates. Participants will learn about health and disability insurance and finances	Sample Topics: Health Insurance, Medicare, Medicaid Disability Insurance Work and Cancer Navigating Finances Estate Planning Cancer-Related Legal Issues	Free, registration required.
**Tools for Role**			
American Cancer Society (ACS)	Resource directory for cancer patients, along with services directly offered by ACS Free rides to treatment Free lodging during treatment		Free
CANCER*care*	List of financial resources, educational workshops, and support groups for cancer patients. Resources: Social work counseling Resource navigation Copay relief Local and national cancer organizations		Free resources Patients must qualify for assistance programs on a need-base basis
Cancer Financial Assistance Coalition	Search tool to find financial or practical help for cancer patients, created by a coalition of cancer support organizations		Free
Family Reach	Organization that provides multiple levels of financial assistance to cancer patients Resource search tool 1:1 resource navigation Financial assistance funds Financial tip sheets		Free
Medicine Assistance Tool	Search tool to find financial assistance for prescriptions.		Free
Needymeds.org	Saving/Coupons on prescriptions, Free/Low-Cost/Sliding Scale Clinics, savings by diagnosis, medical transportation costs, government healthcare programs Free interactive presentation for cancer center and their staff using the GoToMeeting platform. (45 min presentation): covers how to best find assistance on the NeedyMeds website, types of healthcare cost savings assistance available and how to maximize savings with patient’s drug discount card.		Free training on use of website/resources for financial navigators
TailorMed	Program that integrates with electronic medical record to help estimate costs for patients’ specific chemotherapy plans; calculates future drug co-pays and connects patients with programs for assistance.		Paid resource for health systems
Triage Cancer/Triage Health	Free education on the legal and practical issues that may impact individuals diagnosed with cancer and their caregivers; educational material including recorded webinars, quick guides, animated videos, blog, and podcast Legal and Financial Navigation Program: free one-on-one help in the areas of health insurance, disability insurance, employment information, finances, other types of insurance, and estate planning		Free
